# Normalization of Blood Pressure With Spinal Cord Epidural Stimulation After Severe Spinal Cord Injury

**DOI:** 10.3389/fnhum.2018.00083

**Published:** 2018-03-08

**Authors:** Susan J. Harkema, Siqi Wang, Claudia A. Angeli, Yangsheng Chen, Maxwell Boakye, Beatrice Ugiliweneza, Glenn A. Hirsch

**Affiliations:** ^1^Frazier Rehab Institute, Louisville, KY, United States; ^2^Department of Neurological Surgery, Kentucky Spinal Cord Injury Research Center, University of Louisville, Louisville, KY, United States; ^3^Department of Neurosurgery, School of Medicine, University of Louisville, Louisville, KY, United States; ^4^Division of Cardiology, Department of Medicine, University of Louisville School of Medicine, Louisville, KY, United States

**Keywords:** spinal cord injuries, epidural stimulation, cardiovascular system, systolic blood pressure, heart rate

## Abstract

Chronic low blood pressure and orthostatic hypotension remain challenging clinical issues after severe spinal cord injury (SCI), affecting health, rehabilitation, and quality of life. We previously reported that targeted lumbosacral spinal cord epidural stimulation (scES) could promote stand and step functions and restore voluntary movement in patients with chronic motor complete SCI. This study addresses the effects of targeted scES for cardiovascular function (CV-scES) in individuals with severe SCI who suffer from chronic hypotension. We tested the hypothesis that CV-scES can increase resting blood pressure and attenuate chronic hypotension in individuals with chronic cervical SCI. Four research participants with chronic cervical SCI received an implant of a 16-electrode array on the dura (L1–S1 cord segments, T11–L1 vertebrae). Individual-specific CV-scES configurations (anode and cathode electrode selection, voltage, frequency, and pulse width) were identified to maintain systolic blood pressure within targeted normative ranges without skeletal muscle activity of the lower extremities as assessed by electromyography. These individuals completed five 2-h sessions using CV-scES in an upright, seated position during measurement of blood pressure and heart rate. Noninvasive continuous blood pressure was measured from a finger cuff by plethysmograph technique. For each research participant there were statistically significant increases in mean arterial pressure in response to CV-scES that was maintained within normative ranges. This result was reproducible over the five sessions with concomitant decreases or no changes in heart rate using individual-specific CV-scES that was modulated with modest amplitude changes throughout the session. Our study shows that stimulating dorsal lumbosacral spinal cord can effectively and safely activate mechanisms to elevate blood pressures to normal ranges from a chronic hypotensive state in humans with severe SCI with individual-specific CV-scES.

## Introduction

Cardiovascular dysfunction is a leading cause of death in individuals with spinal cord injury (SCI) and has a significant negative impact throughout their lifetime. Individuals with chronic SCI above T6 experience persistent hypotension and bradycardia, with orthostatic hypotension and severe increases in blood pressure during autonomic dysreflexia resulting in drastic daily fluctuations in cardiovascular activity (Wecht and Bauman, 2017). This dysregulation of the autonomic system affects their quality of life by causing discomfort, disrupting their ability to participate in rehabilitation, and interfering with their engagement in daily activities of life. Symptoms of chronic low blood pressure and orthostatic hypotension include fatigue, light-headedness, dizziness, blurred vision, dyspnea, and restlessness associated with cerebral hypo-perfusion (Krassioukov and Claydon, 2006).

The management of chronic hypotension in SCI is a challenging clinical issue as there is no standard or fully successful management strategy. Pharmacological interventions use agents that increase the sympathetic stimulation of the cardiovascular system or increase the blood volume in circulation (Freeman, 2008; Krassioukov et al., 2009). Undesirable side effects of pharmacological interventions and requirement of planning ahead of orthostatic stress have limited their application. Some non-pharmacological interventions have been used with limited success, which include applying external compression to reduce venous pooling in the abdomen or the lower extremities with the elastic compression stocking and abdominal binders (Mathias and Kimber, 1998). Other interventions to improve orthostatic hypotension include functional electrical stimulation (Chao and Cheing, 2005), stand locomotor training (Harkema et al., 2008), and respiratory motor training (Aslan et al., 2016). Although these interventions obtained different degrees of success in reducing the severity of cardiovascular dysfunction, none have fully restored chronic hypotension.

In studies of motor behavior in individuals with motor complete SCI, we observed modulation of cardiovascular parameters when using scES at spinal segments L1–S1 (vertebrae T11–L1) that allows a direct modulation of the spinal neural networks below the level of injury. Targeted scES optimized for standing (stand-scES) enabled four individuals with chronic motor complete SCI to achieve full weight-bearing standing with minimal assistance (Harkema et al., 2011; Rejc et al., 2015, 2017). Targeted scES optimized for voluntary movement (Vol-scES) enabled the same four individuals to perform intentional joint movements (Harkema et al., 2011; Angeli et al., 2014). These studies not only demonstrated effects of scES on altering the excitability of the spinal neural networks to process sensory and supraspinal inputs to improve motor behavior, but also highlighted the importance of optimization of the stimulation configurations, i.e., electrode set, frequency, and intensity, to activate the neuronal pools to achieve targeted tasks and the stimulation parameters were also specific to individuals. During these studies we observed in two individuals who had orthostatic hypotension, blood pressure was elevated and symptoms of orthostatic hypotension were prevented during standing and stepping (unpublished observations).

There have been animal studies (Sato et al., 2002; Yanagiya et al., 2004) and a proof of principle human study in neutrally intact individuals (Yamasaki et al., 2006) showing that scES of the lumbar spinal cord has the potential to ameliorate chronic hypotension. In this study we proposed to use targeted scES optimized for solely cardiovascular function (CV-scES) to provide a unique way to address cardiovascular dysfunction experienced in individuals with SCI. We hypothesized that CV-scES at spinal cord segments L1–S1 with specific targeted configurations can increase resting blood pressure and attenuate orthostatic hypotension in individuals with chronic cervical SCI.

## Materials and Methods

### Research Participants

**FIGURE 1:**
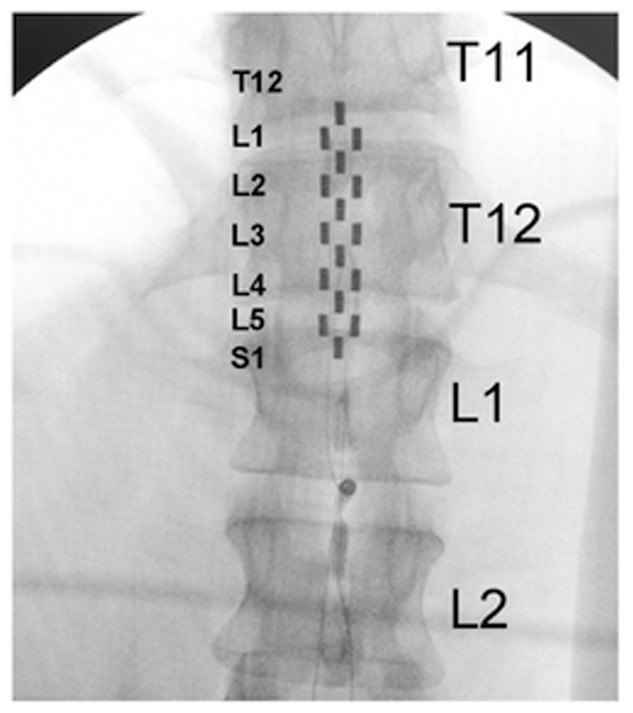
Fluoroscopy showing electrode array location relative to thoracic and lumbar vertebrae. Large letters T11, T12, L1, and L2 identify thoracic and lumbar vertebrae; small letters L1–S1 identify the spinal cord levels estimated by mapping with motor-evoked potentials.

Four research participants with chronic motor complete, cervical SCI were studied (**Table 1**). All individuals were clinically stable, presented with orthostatic hypotension, persistent low resting blood pressure, and routine symptoms of autonomic dysreflexia with no cardiovascular disease unrelated to SCI. The research participants signed an informed consent prior to participating in the study. The study protocol and informed consent were approved by the University of Louisville Institutional Review Board in accordance with the Declaration of Helsinki. A 16-electrode array (5-6-5 Specify, Medtronic, Minneapolis, MN, United States) was implanted to span the spinal cord segments L1–S1 (vertebrae T11–L1) in all research participants (**Figure 1**), as previously described (Harkema et al., 2011; Angeli et al., 2014). The electrode lead was tunneled subcutaneously and connected to the pulse generator (RestoreADVANCED, Medtronic, Minneapolis, MN, United States) placed ventral in the abdomen.

**Table 1:** Characteristics of SCI participants.

Participant ID	Gender	Age (years)	Time since injury (years)	Neuro level of injury	AIS grade	Weight (kg)	Height (cm)
A41	Male	24	7.2	C4	A	84	185
A68	Male	35	3.8	C4	A	60	178
B21	Male	31	7	C4	B	86	183
A80	Female	33	8	C4	A	58	172

*AIS, American spinal injury association Impairment Scale*.

**FIGURE 2:**
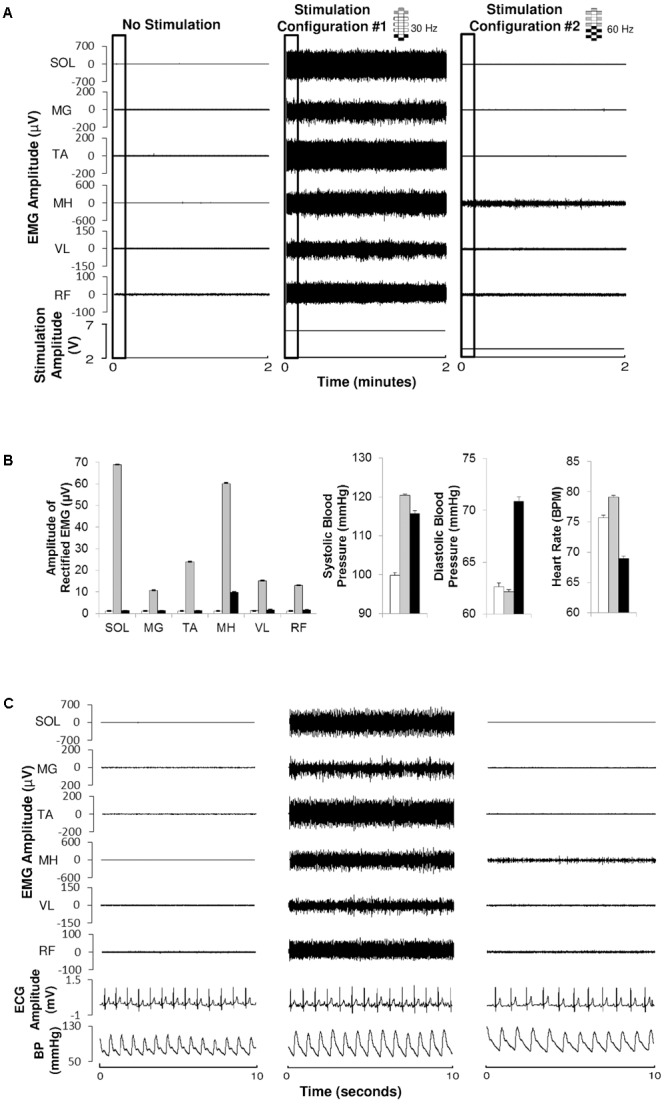
Electromyography, electrocardiography, and blood pressure during different epidural stimulation configurations exemplified in one research participant (B21). **(A)** Electromyography (EMG) of the soleurs (SOL), medial gastrocnemius (MG), tibialis anterior (TA), medial hamstrings (MH), vastus lateralis (VL), and rectus femoris (RF) during 2 min of no stimulation (left), using CV-scES, 30 Hz, Configuration No. 1 (middle) and using CV-scES, 60 Hz, configuration No. 2 (right) in one research participant B21. The electrode selections and stimulation frequency were indicated on the top right of each panel. Gray boxes are cathodes and black boxes are anodes, white boxes are inactive electrodes. **(B)** Amplitude of rectified mean EMG, systolic blood pressure, diastolic blood pressure, and heart rate averaged over 2 min of no stimulation (white bars), configuration No. 1 (gray bars) and configuration No. 2 (black bars) from the data shown in **(A)**. Error bars represent standard error. **(C)** EMG, ECG, and continuous blood pressure in the 10-s windows depicted by the black boxes in **(A)**.

Research participants were resting in a seated position for 2 h with continuous blood pressure and heart rate monitoring. CV-scES configurations (anode and cathode electrode selection, voltage, frequency, and pulse width) were identified to maintain systolic blood pressure within the range of 110–120 mmHg for research participants A41, A68, and B21 and 105–115 mmHg for research participant A80, without skeletal muscle activity of the lower extremities as assessed by electromyography in mapping experiments (**Figure 2**). This usually took two to three sessions that were limited to 2 h. Then five 2-h stimulation sessions occurred where the CV-scES stimulation voltage and frequency would have been adjusted as needed throughout the session in order to target systolic blood pressure within the 110–120 mmHg range (for participants A41, A68, and B21) and 105–115 mmHg range (for participant A80). Pulse width was 450 μs in all sessions for all participants. Diastolic blood pressure and heart rate were also monitored to assure that they were maintained within normative ranges. Resting data were obtained for 15 min before and after the 2-h stimulation period. Data from the first five cardiovascular sessions obtained within 2 weeks are presented.

### Cardiovascular Data Acquisition and Analysis

Noninvasive continuous blood pressure measured from a finger cuff by plethysmographic technique (Finometer Pro, FMS, Amsterdam, Netherlands) was calibrated (Schutte et al., 2003) and recorded continuously in cardiovascular sessions. The continuous data were sampled with 1000 Hz sampling rate with an A/D converter device and computer interface (PowerLab 30/35 Series and LabChart, AD Instruments, Colorado Springs, CO, United States) and stored for offline analysis and were used to guide selection of initial stimulation parameters. Brachial blood pressure measured by oscillometric technique (Carescape V100, GE Healthcare, Milwaukee, WI, United States) was obtained every 2–4 min during the session to calibrate finger blood pressure measurements during the experiments and post-analyses. During offline analysis, beat-to-beat systolic and diastolic blood pressure were calculated as the peaks and nadirs of the finger blood pressure waveform, after finger blood pressure was calibrated to brachial levels and internal calibration artifacts were removed. Mean arterial pressure was calculated as one-third of systolic blood pressure plus two-third of diastolic blood pressure. Heart rate was calculated from the interval between two beats. Beat-to-beat pressures and heart rate were then averaged for every 1 min for statistical analysis. All analyses were performed with customized software in MATLAB (MathWorks, Natick, MA, United States).

### Statistics

Data were summarized graphically using box plots. The box represents the interquartile range values (25th, 50th, and 75th percentiles) and the whiskers extend to non-outliers minimum and maximum data points. Data points 1.5× IQR above the 75th percentile or below the 25th percentile were considered outliers. For each research participant and for the first session, we performed three comparisons for mean arterial pressure, systolic blood pressure, diastolic blood pressure, and heart rate: CV-scES (during stimulation) vs. pre-CV-scES (before stimulation); post-CV-scES (after stimulation) vs. CV-scES (during stimulation); and post-CV-scES (after stimulation) vs. pre-CV-scES (before stimulation). To perform these comparisons, we used linear mixed models on logged values including the experimental phase (pre-CV-scES, CV-scES, and post-CV-scES) as fixed effect and the minutes as random effect nested within period phase, adjusted for the longitudinal nature of the data and the autoregressive nature of the data. Let *y* represent the outcome (mean arterial pressure, systolic or diastolic blood pressure, or heart rate), the equation of the model was specified as follows: log(y_ij_) = β_0_ + β_1_ ∗ time_ij_ + β_2_ phase_j_ + 𝜀_ij_ with 𝜀_ij_ = α_0_ + α_1_ ∗ 𝜀_i-1,j_, where *i* represents the time and *j* represents the experimental phase rank (pre-CV-scES = phase 1, CV-scES = phase 2, and post-CV-scES = phase 3). The three two-by-two comparisons were obtained by constructing linear contrasts from the model.

Each delta mean arterial pressure, delta systolic blood pressure, and delta heart rate value during CV-scES were calculated as the difference of that value and the average of all pre-CV-scES values. These data were not normally distributed and they were also autoregressive (*p*-value of serial correlation < 0.05). To test whether delta mean arterial pressure, delta systolic blood pressure, delta diastolic blood pressure, and delta heart rate are different from 0, we hence used the signed rank test (comparing medians) adjusted for serial correlation using the method of effective sample size (Mitchell et al., 1966; Zwiers and Storch, 1995). Statistical analyses were performed in SAS 9.4 (SAS Institute, Inc., Cary, NC, United States).

### Determination of Initial CV-scES for Each Individual

Each individual completes 3 days of motor mapping of the electrode encompassing voltage response and frequency response curves of local two anode–cathode combinations rostral caudal and left right.

To determine the initial CV-scES configurations, we record EMG activity to identify those that modulate blood pressure but do not elicit motor activity (**Figure 2**). We collected EMG at 2,000 Hz using a 24-channel hard-wired AD board and custom-written acquisition software (Labview, National Instruments, Austin, TX, United States). EMG (MotionLab Systems, Baton Rouge, LA, United States) from the soleus, medial gastrocnemius, tibialis anterior, medial hamstrings, rectus femoris, and vastus lateralis using bipolar surface electrodes with fixed inter electrode distance (Harkema et al., 1997). In addition, two surface electrodes were placed over the paraspinal muscles, symmetrically lateral to the epidural electrode array incision site. These two electrodes were used to record the stimulation artifact from the implanted electrode. EMG data were rectified and high-pass filtered at 40 Hz using Labview software customized by our laboratory. In these experiments we are very often modulating the parameters (anode, cathode selection; frequency and amplitude, number of programs) and the focus is to winnow down to successful CV-scES configurations. The goal is to find a CV-scES configuration that can maintain a relatively stable blood pressure within non-injured defined normal ranges for 2 h without eliciting motor activity.

## Results

### Blood Pressure and Heart Rate Responses to CV-scES

**FIGURE 3:**
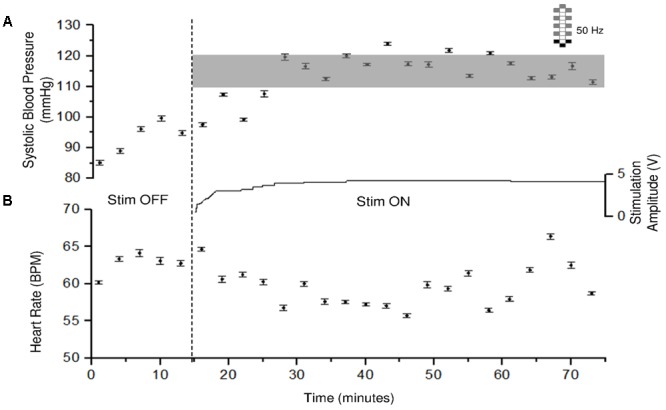
Systolic blood pressure and heart rate responses to CV-scES in one research participant (A68). Systolic blood pressure gradually increased to target range and was maintained during 60 min of activation of stimulation (Stim ON) with minimal adjustment in stimulation amplitude, compared with baseline without stimulation (Stim OFF). Heart rate generally showed an inverse relationship with systolic blood pressure. Solid circles represent sitting systolic blood pressure (mmHg, **top panel**) and heart rate (beats per minute, **bottom panel**) averaged over every 3 min of beat-to-beat values (mean ± SE). Gray shading area represents the target range of systolic blood pressure (110–120 mmHg). Stimulation (Stim) amplitude is shown in the middle and was continuous throughout the 60 min. Frequency was constant at 50 Hz. The vertical dash line indicates the start of stimulation. Electrode configuration is represented on the top right; gray boxes are cathodes, black boxes are anodes, and white boxes are inactive electrodes.

Cardiovascular parameters were normalized consistently in four individuals with chronic cervical SCI using participant-specific CV-scES. Prior to stimulation there is often variability in the systolic blood pressure especially when below 90 mmHg as exemplified in participant A68 (**Figure 3**). This typically occurs is response to yawning or involuntary muscle activation. Systolic blood pressure was gradually increased to the 110–120 or 105–115 mmHg range using the participant-specific CV-scES configuration and could be maintained with minimal modulations in the stimulation amplitude. The stimulation amplitude was increased to increase systolic blood pressure and decreased to reduce systolic blood pressure to maintain the systolic blood pressure within the target range for 60 min. Generally, heart rate was inversely related to systolic blood pressure with some variability over time.

**FIGURE 4:**
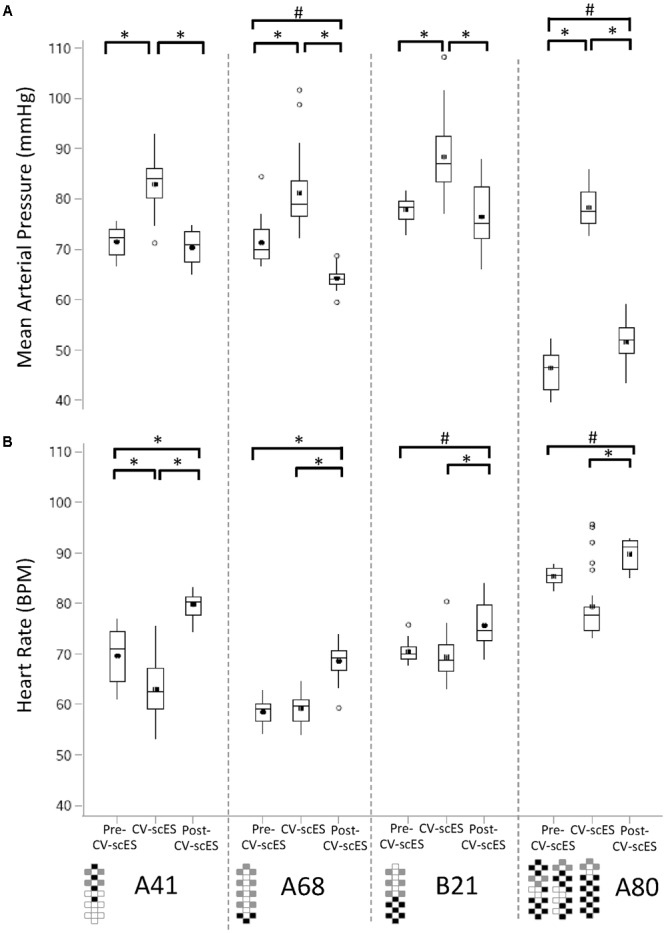
Mean arterial pressure and heart rate response to CV-scES during their first session. **(A)** Mean arterial pressure was significantly higher during CV-scES when compared with Pre-CV-scES and Post-CV-scES in all of the four research participants A41 (left panel), A68 (second panel to the left), B21 (second panel to the right), and A80 (right). Mean arterial pressure was significantly lower during Post-CV-scES compared to Pre-CV-scES in research participant A68 (second panel to the left) and higher in research participant A80 (right panel). **(B)** Heart rate was significantly lower during CV-scES compared to Pre-CV-scES in research participant A41 (left panel). Heart rate was significantly higher during Post-CV-scES when compared with Pre-CV-scES and CV-scES in all four research participants. Data points are 1-min averages of mean arterial pressure and heart rate. Horizontal line, median; solid circle, mean; range of box, interquartile range (25th and 75th percentiles); whiskers, non-outliers maximum and minimum data points; open circles, outliers above or below 1.5× interquartile range. Electrode configurations are represented on the bottom left of the bottom graphs. Gray boxes are cathodes, black boxes are anodes, and white boxes are inactive electrodes. ^∗^*p* < 0.0001; ^#^*p* < 0.003.

**FIGURE 5:**
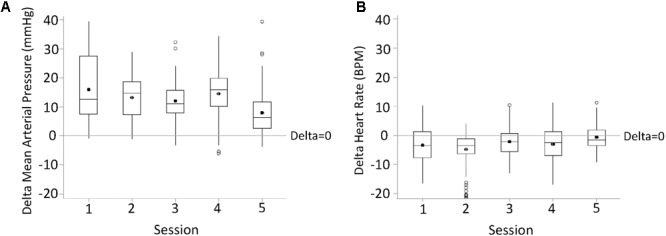
Changes in mean arterial pressure (delta mean arterial pressure, **A**) and heart rate (delta heart rate, **B**) during the first half an hour of active CV-scES from Pre-CV-scES, combined for four research participants averaged for five sessions. Mean arterial pressure increased, and heart rate decreased, from Pre-CV-scES to CV-scES in all five sessions. Data points are 1-min averages of mean arterial pressure and heart rate subtracted from mean values of Pre-CV-scES. Horizontal line, median; solid circle, mean; range of box, interquartile range (25th and 75th percentiles); whiskers, non-outliers maximum and minimum data points; open circles, outliers above or below 1.5× interquartile range. Delta of mean arterial pressure and heart rate of all sessions were significantly different from zero (*p* < 0.05).

For each research participant there were statistically significant increases in mean arterial pressure in response to CV-scES (**Figure 4A**). The mean arterial pressure changes represented concomitant increases in both systolic and diastolic blood pressures as detailed in **Table 2**. This rise in blood pressure returned near or below each participant’s baseline values within 15 min of CV-scES cessation. Three individuals had no significant change in heart rate while A41 had a significant decrease during stimulation. After the CV-scES was turned off heart rate was significantly greater than the baseline for each participant (**Figure 4B**). The average increases in mean arterial pressure during CV-scES for all four participants were reproducible as shown from five sessions over a 2-week period with similar responses among the participants (**Figure 5** and **Table 3**). Individuals reported physical changes during these sessions including: (1) a feeling of alertness or heightened awareness; (2) increased ability to project their voice and carry on conversations; (3) increased capacity to breathe and cough; and (4) overall improved sense of well-being.

**Table 2:** Sitting systolic and diastolic blood pressure before and during CV-scES at the first session day of each participant.

	Systolic blood pressure (mmHg)			Diastolic blood pressure (mmHg)		
Participant ID	Pre-CV-scES	CV-scES	*p*-value	Pre-CV-scES	CV-scES	*p*-value
A41	101 ± 4	111 ± 5	<0.0001	57 ± 2	69 ± 5	<0.0001
A68	97 ± 7	112 ± 11	0.0001	59 ± 4	66 ± 5	<0.0001
B21	106 ± 5	118 ± 9	<0.0001	64 ± 2	74 ± 6	0.0002
A80	65 ± 5	109 ± 8	<0.0001	37 ± 3	63 ± 3	<0.0001

*Data presented as mean ± SD of minute-by-minute time series. p-value, CV-scES vs. Pre-CV-scES.*

**Table 3:** Change in systolic and diastolic blood pressure during CV-scES compared with baseline without CV-scES, combining four participants for each of the five session days.

Session number	Change in systolic blood pressure (mmHg)	*p*-value	Change in diastolic blood pressure (mmHg)	*p*-value
1	20 ± 16	0.0418	14 ± 9	0.0221
2	18 ± 12	0.0184	11 ± 6	0.0026
3	14 ± 14	0.0004	10 ± 8	<0.0001
4	17 ± 10	0.0043	13 ± 7	0.0221
5	12 ± 11	0.008	6 ± 6	0.0031

*Data presented as mean ± SD of minute-by-minute time series. p-value, change in systolic or diastolic blood pressure vs. 0 mmHg.*

### Individual-Specific CV-scES Configurations

**FIGURE 6:**
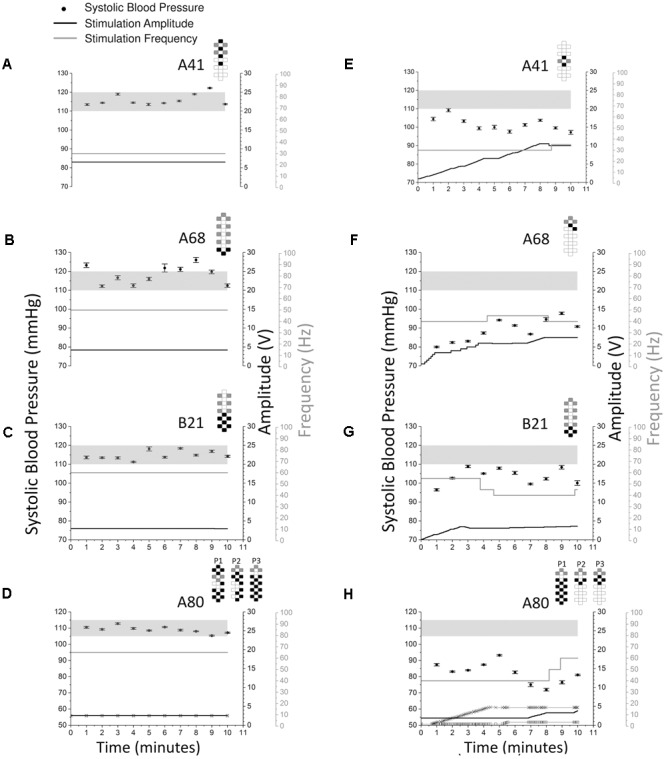
Systolic blood pressure responses to optimal vs. non-optimal stimulation configurations in four research participants (A41: **A** and **E**; A68: **B** and **F**; B21: **C** and **G**; A80: **D** and **H**). Optimal stimulation configuration is important as other configurations failed to increase or maintain systolic blood pressure in the target range. Optimal stimulation configurations are shown on the left **(A–D)** while non-optimal stimulation configurations are shown on the right **(E–H)**. Solid circles represent systolic blood pressure averaged over every 1 min of beat-to-beat values (mean ± SE, left axis). Stimulation was continuous throughout the 10 min, black lines represent stimulation amplitude (right black axis) and gray lines represent stimulation frequency (right gray axis). Participant A80 was stimulated with three programs (**D** and **H**). In **D** and **H**, thin black line with squares represents stimulation amplitude of program No. 1 (P1), thick black line represents stimulation amplitude of program No. 2 (P2), thin black line with crosses represents stimulation amplitude of program No. 3 (P3). The locations of the squares and crosses in **H** indicate amplitude changes. Pulse width was 450 μs in all sessions and experiments for all participants. Gray shading areas represent the target range of systolic blood pressure (110–120 mmHg for research participants A41, A68, and B21; 105–115 mmHg for research participant A80). Electrode configurations are represented on the top right of each graph; gray boxes are cathodes, black boxes are anodes, and white boxes are inactive electrodes.

Each individual required a specific and unique CV-scES configuration (anode–cathode electrode selection, pulse width, frequency, and amplitude) to consistently maintain normalized cardiovascular parameters (**Figure 6**). All four individuals had different configurations that maintained their systolic blood pressure stable within normal limits (**Figures 6A–D**). Also, those configurations were specific and the anode–cathode selection as well as the frequency interaction is important in enabling the consistent control of blood pressure as shown when comparing configurations that were not successful even when continually modulating stimulation amplitude (**Figures 6E–H**).

## Discussion

Persistent hypotension was resolved in four individuals with chronic cervical SCI within normal blood pressure ranges using lumbosacral scES with customized targeted configurations for cardiovascular function (CV-scES). In all four individuals there were significant and reproducible increases in systolic and diastolic blood pressures (**Figures 3, 4** and **Table 2**). Heart rates decreased in one individual and remained relatively stable in the other three during stimulation. These individuals reported that they were invigorated, had better voice projection, were able to communicate more effectively, and could breathe and cough better during these stimulation periods. The normotensive pressures were maintained for 2 h with minor adjustments in amplitude or frequency and without activation of skeletal muscles. This effect was reproducible over multiple sessions (**Figure 5**), and returned to low blood pressures when the stimulation ceased. Our study shows that stimulating dorsal lumbosacral spinal cord can effectively and safely activate mechanisms to elevate blood pressures to a normal range from a chronic hypotensive state in humans with severe SCI with individual-specific CV-scES for a limited time period when the stimulation is present (**Figure 6**).

The eventual activation of sympathetic vasomotor efferents in the lumbosacral spinal cord causing vasoconstriction of the peripheral arteries leading to increase in venous return is a possible mechanism of the increase in blood pressure with CV-scES. The rise in blood pressure then likely stimulated the SCI participants’ intact baroreceptors in the aortic arch and carotid sinus decreasing the heart rate via increased parasympathetic tone maintaining a stabilized system. Yanagiya et al. (2004) provided evidence for controlling both pressor and depressor responses using ES in a model of a “bionic baroreflex” in the epidural space from T12 to L1 in six baroreflex-deafferentiated cats. This study attenuated orthostatic responses using a blood pressure monitor via an indwelling sensor to trigger appropriate scES in response to hypotension. Initially, head-up tilting of these cats led to a decrease in arterial pressure by 59 mmHg in the first 30 s. With use of scES these orthostatic responses significantly attenuated to 8 mmHg at 30 s.

For orthostatic hypotension or resting hypotension, the lower thoracic levels for scES may also be efficacious in raising blood pressure due to the effects of efferent stimulation on the splanchnic vascular bed via sympathetic vasomotor efferents (Hainsworth and Karim, 1976; Minson et al., 1999). Sato et al. (2002) demonstrated similar results in a rat model using an indwelling arterial pressure manometer triggering appropriate electrical stimulation of the vasomotor sympathetic nerve at celiac ganglion in response to hypotension after head-up tilting in 10 rats. Initially, head-up tilting led to a drop in arterial pressure of 52 mmHg at 10 s which was decreased to 15 mmHg after repeat head-up tilting using electrical stimulation. These attenuated hypotensive responses with electrical stimulation were similar to the control experiments with intact native baroreflex function. It is conceivable that splanchnic vasoconstriction also could have contributed to venous return and increased blood pressure in response to CV-scES in our individuals with hypotension secondary to chronic SCI. However, it seems that the site of stimulation is more consistent with the sympathetic fibers described in the Yanagiya et al. (2004) study that the activation of the vasomotor sympathetic efferents from the lumbar cord may be the more prominent mechanism responsible for the effect observed in this study given the similar location and type of response. However, other mechanisms must also be considered, for example, given the dorsal location of the electrode, dorsal fibers that project to intermediolateral columns may reach and influence other levels of the spinal cord, and aspects of the autonomic regulatory system.

A proof of concept human scES trial for the clinical artificial bionic baroreflex system was tested in 21 patients undergoing total knee arthroplasty (Yamasaki et al., 2006). Lower thoracic level scES was effective in attenuating the hypotensive response after rapid onset of depression of blood pressure induced by sudden deflation of thigh tourniquet-simulated central baroreflex failure. The study used percutaneous electrodes with an artificially induced perturbation in blood pressure to provide evidence that the mechanisms described in their animal experiments (Sato et al., 2002; Yanagiya et al., 2004) were translatable to a clinical model. We now demonstrate that targeted and sustained CV-scES in the SCI population of the lumbosacral spinal cord has the potential for providing a similar therapeutic effect specifically in individuals with traumatic SCI.

Other human scES trials for the cardiovascular conditions of heart failure and hypertension had varied responses depending on their pathophysiological state and stimulation site and stimulation configuration (Schultz et al., 2007, 2011; Tse et al., 2015; Zipes et al., 2016). In the case of experimentally induced hypertension using a cold pressor test in individuals with an intact spinal cord who were normotensive, stimulation at higher levels (T1–T2 or T5–T6) had mixed results in attenuating the cardiovascular response (Schultz et al., 2007). A later study in normotensive and hypertensive individuals also with an intact spinal cord did not observe significant changes in mean arterial pressure with epidural stimulation at T5–T6 although they reported some trends and effects on heart rate variability (Schultz et al., 2007). The location and parameters of stimulation were targeted based on animal studies to induce vasodialation (Croom et al., 1997; Tanaka et al., 2001, 2004). The investigators did observe modulation of blood pressure albeit with variability. This may be attributed to individuals with intact spinal cord and cardiovascular regulatory systems can respond and compensate for the stimulatory effects. This may further be supported by the observation that the hypertensive group had more modulation by the stimulation than the normotensive group. These studies differ from our observations in the location of stimulation, the limited parameters of stimulation they were able to assess, and their targeted mechanism of vasodialation. In our study with the permanent implant, we were able to take several hours to identify specific configurations that increased blood pressure that was sub-motor threshold. These studies demonstrated that scES proved to be safe, positioning of the electrode and parameter selection influenced the effect, and the spinal cord stimulation may have clinical implications for hypertension.

Another approach of epidural stimulation in the high thoracic region (T1–T3) in a small cohort human study (Tse et al., 2015) stimulated to improve left ventricular myocardial contractile function and ejection fraction without increasing oxygen consumption based on earlier human studies (Liu et al., 2012). This study showed safety and early feasiblily although a follow-up randomized study of 66 patients did not show a significant difference from a comparison group of a cardioverter-defibrillator group with left ventricular ejection fraction as the primary outcome measure (Zipes et al., 2016). Interestingly to note in the larger cohort is that the study was stopped prior to the planned enrollment of 195 subjects and statistical significance between the two groups was not sufficiently powered to see a significant difference between the two groups. Also, spinal cord stimulation was arbritrarily chosen from animal studies and on the basis of those used for patients with intractable angina. Physiological surrogates were not used for each individual to identify stimulation parameters. We found that to maintain normative blood pressures each person required unique CV-scES parameters and it took mapping for several hours using physiological measures to identify them. These studies concluded that the spinal cord stimulation was safe; however, there is significant variability with the site of stimulation, the pathophysiology, as the configuration selection all clearly will impact the outcome and need to be carefully considered when designing clinical studies and treatments.

The current study, when also taken in context with other recent studies of epidural stimulation, demonstrates that individuals diagnosed with motor complete SCI may have significant potential for recovery. In the presence of epidural stimulation of spinal networks there now is evidence that individuals who have been diagnosed with motor complete SCI can regain volitional movement (Angeli et al., 2014) and the ability to stand (Rejc et al., 2015, 2017), and in this study we show that they can potentially resolve another devastating consequence of SCI, cardiovascular dysfunction. These reversals of the repercussions of SCI in those with the most severe injuries, those determined that there is the most isolation from supraspinal input, provide us with the ability to gain insights to the infrastructure and capacity for plasticity of the human spinal circuitry (Grillner and Wallen, 2004). A key factor in these studies is that each motor behavior and physiological response has its own unique stimulation configuration indicating that the successful execution of the response is dependent on the appropriate excitability of the spinal network. Each individual also has a unique configuration and that may have many contributing factors including placement of electrode, differences among injuries, time since injury, pharmaceuticals, and levels of activity.

Epidural stimulation directly activated the spinal circuitry and immediately improved blood pressure in these individuals with SCI. There are other non-invasive modalities that can access the spinal circuitry such as transcutaneous stimulation (Gerasimenko et al., 2015, 2016). Locomotor training is an activity-based therapy that activates the neuromuscular system below the level of injury (Harkema et al., 1997; Beres-Jones and Harkema, 2004) and has been shown to improve blood pressure in individuals with incomplete SCI (Ditor et al., 2005). Both of these target the spinal circuitry that is available after SCI and also is a significant target for therapy (Harkema et al., 2012a,b). These findings provide a proof of principle that there may be many avenues available for significant recovery for individuals who live with paralysis that have not yet been fully taken advantage of in clinical environments.

The availability of implantable scES devices provides a novel treatment for chronic SCI patients to improve symptomatic resting and orthostatic hypotension. Longer term studies are needed to assess cardiovascular effects of CV-scES on myocardial structure and function, vascular biology, and clinical outcomes in this population. Development of vascular pressure sensing devices that can activate appropriate CV-scES as a closed feedback system is essential for home and community use and could ameliorate this major impairment to quality of life in the SCI population. These findings are important not only to the chronic SCI population with substantial morbidity and mortality related to excess cardiovascular disease including extreme instability and liability of blood pressure control, but also to other disease states with significant morbidity from orthostatic hypotension such as multiple system atrophy (previously called the Shy-Drager syndrome) or baroreceptor-deafferentiated states such as those that occurs after carotid sinus disruption from trauma, vascular surgery, or tumor involvement. Other dietary treatments such as salt loading or pharmacologic therapy with adrenergic agonists do not consistently prevent orthostatic hypotension and can lead to supine hypertension. CV-scES provides a potential unique treatment option for SCI patients to normalize blood pressure and alleviates the adverse impacts of chronic low blood pressure and orthostatic hypotension on physical, emotional, and social well-being (Carlozzi et al., 2013).

## Limitations and Future Studies

This study was limited to four individuals and needs to be repeated in a larger number of people with chronic SCI over longer periods of time to understand the broader application and to continue to understand the human spinal circuitry and the capacity of recovery after SCI. Further analyses of mechanisms focused on heart rate and blood pressure variability and post-stimulation effects are needed. Larger, prolonged studies of this potential approach as a therapy are warranted to assess additional cardiovascular effects and whether it can improve symptoms or outcomes in other diseases characterized by low blood pressure and orthostatic hypotension. Closed-looped control systems need to be developed to allow monitoring and usability in real-life environments and in varied situations that will induce challenges to the cardiovascular system. Long-term effects of scES on autonomic dysreflexia and the respiratory system both related to these cardiovascular deficits also need to be studied.

## Author Contributions

SH and CA designed the study. GH and MB provided the medical oversight. MB performed the implant procedure with SH, CA, and YC, providing the physiological data during the surgical procedure. SH identified the configurations for the cardiovascular stimulation, provided oversight for data acquisition and analyses, and performed cardiovascular sessions. SW and BU performed the data acquisition and analyses. All authors were involved in data interpretation and manuscript preparation.

## Conflict of Interest Statement

The authors declare that the research was conducted in the absence of any commercial or financial relationships that could be construed as a potential conflict of interest.

## Abbreviations

AIS: American spinal cord injury association impairment scale
CV-scES: spinal cord epidural stimulation targeted specifically for cardiovascular function
post-CV-scES: time period following epidural stimulation targeted specifically for cardiovascular function
pre-CV-scES: time period prior to beginning epidural stimulation targeted specifically for cardiovascular function
scES: spinal cord epidural stimulation
SCI: spinal cord injury
stand-scES: standing targeted spinal cord epidural stimulation
Vol-scES: voluntary movement targeted spinal cord epidural stimulation
